# Inhibition of spinal bone formation in AS: 10 years after comparing adalimumab to OASIS

**DOI:** 10.1186/s13075-019-2045-1

**Published:** 2019-11-06

**Authors:** Désirée van der Heijde, Robert Landewé

**Affiliations:** 1 Department of Rheumatology, Leiden University Medical Center, P.O. Box 9600, 2300 RC Leiden, The Netherlands; 2 Amsterdam University Medical Center (ARC), Amsterdam, The Netherlands; 3 Zuyderland Medical Center, Heerlen, The Netherlands

**Keywords:** Ankylosing spondylitis, Structural damage, TNF inhibitor

## Abstract

A decade has passed since the publication on the comparison of the effect of adalimumab with data from a historic cohort on the progression of structural damage in the spine of patients with ankylosing spondylitis (AS). No effect could be observed, and currently, there is still no definite proof that TNF inhibitors (TNFi) inhibit spinal structural damage. The findings of the publication are discussed within the context of the time at the publication and new developments.

In 2009, we published about the effects of 2-year treatment with the TNF inhibitor adalimumab on spinal radiographic progression in patients with ankylosing spondylitis (AS) [1]. The remarkable and somewhat unusual comparator group was formed by a historic cohort of patients with AS treated with NSAIDs (nonsteroidal anti-inflammatory drugs) and csDMARDs (conventional synthetic disease-modifying antirheumatic drugs): the OASIS (Outcome in AS International Study) cohort [2]. The scoring method was the mSASSS (modified Stoke AS Spinal Score), a method using conventional radiography [3]. There was no slowing of radiographic progression by adalimumab, neither in the full OASIS cohort nor in the subgroup of patients that retrospectively should have fulfilled the entry criteria for the adalimumab trials. Many people were disappointed by these “unexpected” results, even though similar results had been shown for etanercept and infliximab [4, 5]. Still, people kept hope that a different monoclonal antibody against TNF should prove inhibition of bone proliferation, likely in view of inhibitory effects of these drugs in RA (rheumatoid arthritis) and PsA (Psoriatic Arthritis). But, as shown in the paper, the observed progression in the three groups of patients treated with a TNFi and the three independent reads of the same OASIS films showed very similar mean progression rates of 0.8 to 1.0 (SD 2.6–3.3) units. Obviously, the reading by the mSASSS method was very robust, as each assessment was performed by different reader pairs and yielded consistent 2-year rates of progression in four cohorts of patients (i.e., three trials and OASIS).

The different reactions to these disappointing results could be distinguished in two opposing opinions: (1) “there must be an effect, so if you do not see it there will be good reasons to explain this” and (2) “the pathophysiology of the underlying bone processes in RA (bone-destruction) and AS (bone formation) differ diametrically, which explains the lack of inhibitory effect on spinal bone formation.” Some hypotheses supporting the first view (“you missed the effect”) included differences in severity (disease activity and prognostic factors) between the patients treated with adalimumab and those from OASIS, a too short period of depression of inflammation in a 2-year trial, a too late start in the disease to prevent reparative processes leading to bone formation, and a too insensitive outcome measure (mSASSS). The opponents of the “expected effect” hypothesis argued that bone formation often develops at sites without inflammation, that the Wnt signaling pathway is more involved in bone formation than the TNF pathway, and even that extra bone formation could occur when TNF is inhibited [6, 7].

## What have we learned in the subsequent decade?

A very important finding was the formal proof of a longitudinal relationship between (increase in) disease activity (assessed as ASDAS (AS Disease Activity Score) and subsequent (increase in) mSASSS [8]. Another important finding was that the presence of inflammation at a vertebral corner, as seen on MRI, increases the probability of the formation of a syndesmophyte at that site on a radiograph 2 years later. It was made likely that this happens at those corners in which inflammation disappears and is replaced by fatty infiltration, but not at corners with persistent inflammation [9]. Still, the majority of syndesmophytes develop at corners without (observed) inflammation on MRI. The robust relationship between disease activity and MRI inflammation on one side and structural damage on the other side adds to the likelihood that a TNFi-induced suppression of disease activity (either measured clinically as a decrease of ASDAS or on sequential spinal MRIs) may also lead to inhibition of spinal bone formation.

Several cohort data have been published addressing the effects of TNFi [10–12]. However, cohort data suffer from various issues (such as confounding by indication, selection of patients with available radiographs, radiographs not taken in relation to start of TNFi, different intervals between radiographs, insufficient information about other treatments) [13]. Sophisticated statistical modeling aims to overcome these issues, but many assumptions have to be made and statistics do not adjust for unknown information. Notwithstanding these issues, several cohorts suggest an inhibitory effect of TNFi on radiographic progression, with various levels of persuasion. These effects are especially seen with longer follow-up (4 instead of 2 years), when disease activity is reduced or when TNFi is combined with NSAIDs [10, 11].

Despite all technical progress in the field of imaging, conventional radiography of the spine and the mSASSS as a scoring method have survived the “ravages of time” [13]. Still, they have important disadvantages such as insufficient resolution, inclusion of only half the spine (because of overprojection by pulmonary tissue), and poor sensitivity to change; a follow-up of at least 2 years is needed to demonstrate sufficient progression. Recently, a new scoring method exploiting images obtained by *low-dose* CT scans was presented [14, 15]. The resolution of CT has always been superior to that of conventional radiography, but widespread application (e.g., in trials) was limited by prohibitory radiation dose levels. Software adaptations allowed the acquisition of proper quality CT images using far lower doses of radiation, and CT has thus become a feasible alternative. Most importantly, low-dose CT includes the whole spine and has in the meantime already proven far higher sensitivity to change. These developments may allow studies with lower numbers of patients and a shorter follow-up but still sufficient statistical power to demonstrate a difference in bone formation if it really exists.

Comparisons of contemporary trial populations with historical cohorts without b(biological)DMARD use such as OASIS have become less attractive since contemporary trials now likely include less severe patients than in the early years of TNFi trials. Having said that, since new treatments for AS, such as IL17i, have become available recently, it will now be possible and ethically justifiable to perform a head-to-head trial with two active treatments (i.e., TNFi vs. IL17i) for a period of 2 years. Such a trial may provide an answer to the question if bDMARDs inhibit bone proliferation in AS, but only if one of both treatments has a larger impact on structural damage progression than the other treatment. If both classes of bDMARDs reduce progression of bone formation equally well, this matter will remain concealed, but with the advent of additional new treatments, the likelihood of a differential effect on syndesmophyte formation will increase. It may still take another decade to get the final answer to the question if there is really a treatment for AS that reduces spinal bone proliferation and bamboo spine formation.

## Data Availability

Not applicable

## Abbreviations

AS: Ankylosing spondylitis
ASDAS: AS Disease Activity Score
csDMARDs: Conventional synthetic disease-modifying antirheumatic drugs
mSASSS: Modified Stoke AS Spinal Score
NSAIDs: Nonsteroidal anti-inflammatory drugs
OASIS: Outcome in AS International Study
PsA: Psoriatic arthritis
RA: Rheumatoid arthritis
